# Supplementary material for: Urine Di-(2-ethylhexyl) Phthalate Metabolites Are Independently Related to Body Fluid Status in Adults: Results from a U.S. Nationally Representative Survey
Source: Int J Environ Res Public Health. 2022 Jun 7;19(12):6964. doi: 10.3390/ijerph19126964 (PMC9222572; doi:10.3390/ijerph19126964)
Supplement: Supplementary file 1 [file ijerph-19-06964-s001.zip › ijerph-1737989-supplementary.pdf]

Urine Di-(2-ethylhexyl) Phthalate Metabolites Are Independently Related to Body Fluid Status in Adults: Results from a U.S. Nationally Representative Survey

Wei-Jie Wang, Chia-Sung Wang, Chi-Kang Wang, An-Ming Yang and Chien-Yu Lin

Supplementary material

Urine DEHP metabolites:

In NHANES 2003-2004, urine DEHP metabolites including mono (2-ethylhexyl) phthalate (MEHP), mono(2-ethyl-5-oxohexyl) phthalate (MEOHP), mono(2-ethyl-5-hydroxyhexyl) phthalate (MEHHP), and mono(2-ethyl-5-carboxypentyl) phthalate (MECPP) were tested by using high performance liquid chromatography-electrospray ionization-tandem mass spectrometry. Urine samples are processed using enzymatic deconjugation of the glucuronidated phthalate monoesters followed by on-line solid phase extraction coupled with reversed phase HPLC-ESI-MS/MS. Assay precision is improved by incorporating isotopically-labeled internal standards for each of the phthalate metabolites. For concentrations below detection limits, a value was assigned by NHANES. We used this value in our analyses. The detailed method is available at NHANES website[1].

Body Measures:

The body measurement assessments performed on survey participants varied

according to the participants' ages. Body weight data for individuals who had limb amputations were excluded from the release file. This data set includes body measurements for women who were pregnant at the time of the exam. BMI was calculated as weight in kilograms divided by height in meters squared. The NHANES anthropometry protocol also included triceps and subscapular skinfold measures for participants aged 2 months and older. Both types of skinfolds were measured in millimeters using the Holtain skinfold caliper. The body measurement techniques used in the survey are illustrated in the NHANES III Anthropometric Procedures Video. For the procedures relevant to this component, please refer to the NHANES website to obtain the Survey Operations Manual, Consent Documents, and brochures [2].

Bioelectrical Impedance Analysis (BIA):

This examination was conducted in eligible survey participants 8-49 years of age. Pregnant females were not eligible for the BIA examination. Other reasons for exclusion included amputations other than fingers or toes, artificial joints, pins, plates, or other types of metal objects in the body, pacemakers or automatic defibrillators, coronary stents or metal suture material in the heart, and weight over 300 pounds (limitation of the examination table).

The NHANES BIA data were collected with a HYDRA ECF/ICF Bio-Impedance

Spectrum Analyzer (Model 4200, Xitron Technologies, Inc., San Diego, California, USA). The multi-frequency analyzer uses a full 12-bit digital signal processing technique to measure impedance at 50 frequencies logarithmically spaced from 5 KHz to 1 MHz (HYDRA, 1997). Bioelectrical impedance analysis measures the electrical impedance of body tissues and can be used to assess total body water, fat mass and fat-free body mass. A small alternating current was passed through surface electrodes placed on the right hand and foot, and the impedance to the current flow was measured by different electrodes placed adjacent to the injection electrodes. The voltage drop between electrodes provided a measure of impedance, or opposition to the flow of the electric current. Detailed information is available at the NHANES website [3].

Covariates:

We considered age, gender, race/ethnicity, education level, household income, smoking status, caffeine intake, total energy intake, total protein intake, total sugar intake, total carbohydrate intake, total saturated fatty acid intake, metabolic equivalent intensity level for activity to be potential confounders of body composition. Data were collected at all study sites by trained personnel using standardized procedures. Sociodemographic information, such as age, gender, race/ethnicity, education level, and household income, was recorded during the household interview. Smoking status

was categorized as active smoker, exposed to environmental tobacco smoke (ETS), or non-exposed by the smoking questionnaire and serum cotinine levels, as described previously [4]. Serum cotinine was measured by isotope dilution-high performance liquid chromatography / atmospheric pressure chemical ionization tandem mass spectrometry. Active smokers were defined as those with cotinine levels > 15 ng/mL or those who reported currently smoking every day or on some days. Those with serum cotinine levels that were detectable but ≦ 15 ng/mL and who did not report current smoking were considered to be exposed to environmental tobacco smoke. Cotinine levels of < 0.015 ng/mL were below the detection limit. Those with undetectable serum cotinine levels, no reported smoking in the home, and no self-reported smoking were considered to be non-exposed. The dietary intake data were used to estimate the types and amounts of foods and beverages consumed during the 24-hour period prior to the interview (midnight to midnight) and to estimate intake of energy, nutrients, and other food components from those foods and beverages. Two days of intake data were collected for each participant. The first day data were collected in the Mobile Examination Center, and the second day data were collected by telephone 3 to 10 days later. We averaged the caffeine intake, total energy intake, total protein intake, total sugar intake, total carbohydrate intake, and total saturated fatty acid intake from the two days as covariates in this study. Physical Activity was

assessed in each NHANES participant by a physical activity questionnaire which was based on the daily activities, leisure time activities, and sedentary activities at home. All survey participants 12 years of age or older were eligible for information about specific leisure-time activities. Metabolic equivalent scores for the activities were obtained from the appropriate reference and through personal communication with the author [5].

References

1. 2003–2004 Data Documentation, Codebook, and Frequencies: Phthalates—Urine. Available online: https://wwwn.cdc.gov/Nchs/Nhanes/2003-2004/L24PH_C.htm (accessed on 10 December 2021).
2. 2003–2004 Data Documentation, Codebook, and Frequencies: Body Measures. Available online: http://wwwn.cdc.gov/Nchs/Nhanes/2003-2004/BMX_C.htm (accessed on 10 December 2021).
3. 2003–2004 Data Documentation, Codebook, and Frequencies: Bioelectrical Impedance Analysis. Available online: http://wwwn.cdc.gov/Nchs/Nhanes/2003-2004/BIX_C.htm (accessed on 10 December 2021).
4. Weitzman, M.; Cook, S.; Auinger, P.; Florin, T.A.; Daniels, S.; Nguyen, M.; Winickoff, J.P. Tobacco smoke exposure is associated with the metabolic syndrome in adolescents. *Circulation* **2005**, *112*, 862–869.
5. 2003–2004 Data Documentation, Codebook, and Frequencies: Physical Activity. Available online: https://wwwn.cdc.gov/Nchs/Nhanes/2003-2004/PAQ_C.htm#Appendix_1._Suggested_MET_Scores (accessed on 10 December 2021).
